# Clinical efficacy and metabolomics profiling of dachaihu decoction for patients with septic liver injury: a randomized controlled trial

**DOI:** 10.3389/fphar.2025.1671732

**Published:** 2025-11-25

**Authors:** Zhen Yang, Xingyu Kao, Tianwei Zhu, Junna Lei, Na Huang, Jingli Chen, Mingfeng He, Qi Tang, Zhangrong Liang

**Affiliations:** 1 The Eighth Clinical Medical College of Guangzhou University of Chinese Medicine, Foshan, China; 2 Department of Emergency Medicine, Foshan Hospital of Traditional Chinese Medicine, Foshan, China; 3 Department of General Practice, Foshan Hospital of Traditional Chinese Medicine, Foshan, China

**Keywords:** septic liver injury, dachaihu decoction, randomized controlled trial, traditional Chinese medicine, metabolomics, bile acid metabolism

## Abstract

**Background:**

Septic liver injury (SLI) is a life-threatening complication of sepsis with limited therapeutic options. The clinical efficacy and safety of Dachaihu Decoction (DCHD) in SLI remain to be elucidated.

**Methods and Design:**

A prospective, single-center, single-blind, randomized, and placebo-controlled clinical trial was conducted. Patients in the DCHD group received DCHD twice a day for five consecutive days on the basis of sepsis bundle, while patients in the placebo group were administered a placebo at the same dosage. Primary outcomes included: (1) liver function indices: alanine transaminase (ALT), aspartate transaminase (AST) and total bilirubin (TBil); (2) Sequential Organ Failure Assessment (SOFA) and Acute Physiology and Chronic Health Evaluation II (APACHE II) scores; (3) 28-day all-cause mortality. Secondary outcomes included the evaluation of several clinical parameters: (1) infection indicators; (2) coagulation indicators; (3) gastrointestinal function indicator; (4) metabolic and respiratory function indicators. Subsequently, we employed Liquid Chromatography-Tandem Mass Spectrometry (LC-MS/MS) to characterize the serum metabolomics profiling of two groups of patients.

**Results:**

DCHD significantly reduced TBil (−22.50 (interquartile range, IQR, −37.20, −8.10) *vs.* −3.30 (−17.16, 12.40), *p* < 0.001), SOFA score (−2.46 ± 2.84 *vs.* −1.11 ± 2.71, *p =* 0.047), APACHE II score (−5 (IQR, −5, −2) *vs.* −2 (−5, 2), *p =* 0.034), and Oxygenation Index (OI) (29.71 ± 74.76 *vs.* −15.16 ± 108.51, *p =* 0.048). However, no statistically significant difference in 28-day all-cause mortality was found between the DCHD and the placebo groups (7 (20.0%) *vs.* 9 (25.7%), *p =* 0.569). Additionally, our study demonstrates that DCHD ameliorates systemic infection, coagulation function, gastrointestinal function, and metabolic function in patients to a certain extent, and no severe side effects were reported. Metabolomics analysis reveals that Wogonin, Wogonoside, Cholic acid, and Glycocholic acid are representative differential metabolites, and bile acid metabolism may be the core metabolic pathway.

**Conclusion:**

As an adjunctive therapy, DCHD demonstrates safety and efficacy in the treatment of SLI, particularly cholestatic hepatic dysfunction, which may be intimately linked to its modulation of bile acid metabolism.

**Clinical Trial Registration:**

http://itmctr.ccebtcm.org.cn, identifier ITMCTR2025000095.

## 1 Introduction

Sepsis, a life-threatening systemic disorder characterized by dysregulated host responses to infection, continues to impose a substantial global health burden as a leading cause of intensive care unit (ICU) mortality (Rudd et al., 2020; Singer et al., 2016). Recent epidemiological estimates indicate approximately 48.9 million annual sepsis cases worldwide, accounting for 11 million deaths (Rudd et al., 2020). Septic liver injury (SLI) is a common and serious complication in the development of sepsis, approximately 34%–46% of patients with sepsis develop liver injury as reported and significantly exacerbates poor clinical outcomes (Chen et al., 2024). The pathogenesis of SLI involves a complex interplay of maladaptive immune activation, oxidative stress-mediated cellular damage, mitochondrial dysfunction, and dysregulated apoptotic pathways (Wang et al., 2014). Cholestasis represents the most prevalent clinical manifestation of SLI, characterized by a marked elevation in total bilirubin (TBil) (Jenniskens et al., 2016). Clinical evidence demonstrates that hepatic involvement in sepsis portends a 1.6- to 2.1-fold increase in mortality risk compared to septic patients without liver injury, with fatal outcomes observed in 54%–68% of cases according to multicenter cohort studies (Chen et al., 2024; Savio et al., 2017). Despite advancements in sepsis management strategies, including early antimicrobial therapy and hemodynamic optimization, the development of specific and highly effective hepatoprotective agents remains elusive (Xu et al., 2025).

Traditional Chinese Medicine (TCM) exhibits systemic therapeutic advantages in managing infection-related disorders, particularly through multi-target modulation of host immunity and inflammation (Zou et al., 2023). For instance, TCM formula Wenqingyin has demonstrated therapeutic efficacy in a lipopolysaccharide (LPS) induced SLI mouse model, exerting its effects by simultaneously suppressing pro-inflammatory cytokines (e.g., IL-6, TNF-α) and enhancing Nrf2-mediated antioxidant pathways (Xie et al., 2023). Notably, SLI belongs to the category of TCM “epidemic” diseases, and “heat, poison, stasis, and deficiency” are the pathological characteristics of SLI(Wu et al., 2021). Our research team has long been committed to the clinical and mechanistic investigation of sepsis. We innovatively proposed that the combination of Shaoyang and Yangming Syndrome Complex constitutes the core pathogenesis of sepsis (Huang et al., 2023), which is consistent with the TCM pathogenesis of SLI “intense heat-toxin accumulation in the liver-gallbladder system (Shaoyang) with concurrent excess stagnation and obstruction in the stomach-intestine system (Yangming)”. Therefore, dredging fu-organs to soothe the liver (*tong fu shu gan*) combined with clearing heat-toxin (*qing re jie du*) constitutes the core therapeutic principle for SLI. The metabolic processing of Chinese herbal medicine within the body constitutes a pivotal stage in their therapeutic efficacy. Serum metabolomics serves as a powerful tool for dynamically tracking the distribution and metabolic dynamics of bioactive components. This approach provides critical evidence for elucidating the molecular mechanisms by which multi-component TCM formulae synergistically modulate metabolic homeostasis (Wang et al., 2017).

Originating from the Treatise on Cold Damage and Miscellaneous Diseases (Shanghan Zabing Lun), Dachaihu Decoction (DCHD) is a classical formula rooted in the “Shaoyang-Yangming synergy” theory, designed to harmonize the liver-gallbladder system, purge pathogenic heat, and restore gastrointestinal motility. DCHD consists of *Bupleuri Radix* (Chaihu), *Scutellariae Radix* (Huangqin), *Rhei Radix et Rhizoma* (Dahuang), *Aurantii Fructus Immaturus* (Zhishi), *Paeoniae Radix Alba* (Baishao), *Pinelliae Rhizoma* (Banxia), *Zingiberis Rhizoma Recens* (Shengjiang), and *Jujubae Fructus* (Dazao) in a weight ratio of 5:3:2:3:3:3:5:4, which work together to exert the effects of dredging the fu organs and draining heat (*tong fu xie re*), as well as soothing the liver and detoxifying pathogens (*shu gan jie du*) (Huang et al., 2025a). Our previous studies have demonstrated DCHD capacity to suppress inflammatory cascades, mitigate oxidative stress, enhance intestinal barrier function, and exert multi-organ protective effects targeting the pulmonary, intestinal, hepatic, and renal systems *in vitro* and *in vivo* (Huang et al., 2023; Huang et al., 2025b; Yang et al., 2025). Moreover, Accumulating studies have demonstrated the significant therapeutic efficacy of DCHD in managing inflammatory disorders and hepatobiliary diseases, including non-alcoholic fatty liver disease (Cui et al., 2020), cholecystitis (Liu et al., 2024), and pancreatitis (Li et al., 2025), and these therapeutic benefits are mediated through its regulatory effects on pathophysiological processes such as inflammatory cell infiltration and lipid metabolic homeostasis. In contrast, certain potential hepatoprotective agents for sepsis act through more targeted mechanisms. For instance, N-acetylcysteine primarily replenishes glutathione to combat oxidative stress and quell the cytokine storm, whereas ursodeoxycholic acid replaces toxic bile acids to mitigate cholestasis and stabilize hepatocyte membranes (Li et al., 2020; Szakmany et al., 2012). Nevertheless, clinical studies on TCM for SLI are scarce, particularly high-quality randomized controlled trial.

Therefore, we conducted a prospective, single-center, single-blind, randomized, and placebo-controlled clinical trial to confirm the efficacy and safety of DCHD as adjunctive therapy in patients with SLI. Meanwhile, untargeted metabolomics was employed to investigate metabolic alterations in SLI patients following DCHD treatment, and to screen differential metabolites (DMs) and related metabolic pathways.

## 2 Methods

### 2.1 Trial design

This trial was a prospective, single-center, single-blind randomized controlled clinical trial conducted from January 2024 to January 2025 in the ICU, Emergency ICU (EICU), and Neurological ICU (NICU) of Foshan Hospital of TCM, a tertiary care hospital (Level IIIA hospital). This trial was conducted in accordance with the principles of Good Clinical Practice guidelines and the Declaration of Helsinki (World Medical Association, 2013), and was approved by the Medical Ethics Committee of Foshan Hospital of TCM (ethical approval number: KY [2024]010) (Supplementary file S1). This trial was also registered on the International Traditional Medicine Clinical Trial Registry (http://itmctr.ccebtcm.org.cn, registration number: ITMCTR2025000095, Registered on 13 January 2025). The ethics committee of our hospital is responsible for supervising the trial, and the participants included in the study have signed a written informed consent. Our trial followed CONSORT guidelines to ensure rigor and reliability of the study (Supplementary file S2) (Butcher et al., 2022).

### 2.2 Participants

The diagnostic criteria for SLI: ① Diagnostic criteria for sepsis: evidence of infection and Sequential Organ Failure Assessment (SOFA) score ≥2 (Singer et al., 2016); ② Serum alanine transaminase (ALT) > 2 times the upper limit of normal value (The normal ALT level in our hospital is 50 U/L); ③ TBil ≥34.2 μmol/L (2 mg/dL); ④ International normalized ratio (INR) > 1.5. Two of ②-④ must be met in addition to ① (Dellinger et al., 2013; Miao et al., 2023). (2) Age ≥18 years; (3) Signed the informed consent. The exclusion criteria were: (1) Patients with liver injury not caused by sepsis; (2) Patients with pre-existing liver diseases, including viral hepatitis, liver abscess, autoimmune liver diseases, cirrhosis, or hepatocellular carcinoma; (3) Patients with a history of allergy to herbal medicine; (4) Patients ineligible for enteral feeding; (5) Patients concurrently participating in other clinical trials; (6) Pregnant or lactating patients; (7) Patients with incomplete essential data; (8) Patients presenting with diarrhea, hypothermia, preshock, or shock; (9) Patients with intervention duration <5 days or who died within 5 days post-enrollment.

### 2.3 Sample size

The sample size was estimated using Power Analysis and Sample Size (PASS, version 15.0, NCSS). Under the “Proportions” module, the subcategory “Two Independent Proportions” was selected, followed by “Superiority by a Margin.” The analysis utilized the “Superiority by a Margin Tests for the Difference Between Two Proportions” method. Parameters were set as follows: (1) α (two-sided significance level) = 0.05; (2) β (type II error rate) = 0.15, corresponding to a statistical power of 85%; (3) Allocation ratio = 1:1; (4) Expected efficacy rates: 86.7% for the treatment group vs. 43.3% (You et al., 2018) for the control group, superiority margin = 12%. The calculation yielded a minimum requirement of 33 subjects per group. To account for a 20% anticipated dropout rate, the final adjusted sample size was 42 patients per group.

### 2.4 Randomization and blinding

A randomization schedule was generated using SPSS 27.0 software (IBM, Armonk, New York, United States). Eligible patients were sequentially randomized based on their order of admission and assigned to either the DCHD group or placebo group using a 1:1 ratio. Random numbers and group assignments were sealed in opaque, sequentially numbered envelopes to ensure allocation concealment. Two research assistants uninvolved in clinical care or outcome assessment were responsible for opening the envelopes and assigning eligible participants according to the predefined randomization protocol (Supplementary file S3).

Due to the critical clinical status of septic patients and their potential for sudden clinical deterioration, blinding of investigators or healthcare providers was not implemented in this trial. However, blinding was rigorously maintained for participants, outcome assessors, data collectors, and analysts to minimize bias.

### 2.5 Interventions

All patients with SLI received the standard treatment protocol adheres to the 2021 Surviving Sepsis Campaign Guidelines and American Association for the Study of Liver Diseases (AASLD) recommendations, which encompassed the following key components: timely antimicrobial therapy, early goal-directed fluid resuscitation, vasoactive agents, organ function support, anticoagulation therapy, hepatoprotective agents, and nutritional support (Evans et al., 2021; Karvellas et al., 2024). Patients in the DCHD treatment group received DCHD intervention for 5 consecutive days. The components and dosage of the DCHD are detailed in Table 1. In order to minimize the batch effect, the herbal formulation employed in this trial was sourced exclusively from a single production batch and followed the ConPhyMP checklist (Supplementary file S4). Quality assurance procedures included verification and preparation by licensed pharmacists at the Department of Pharmacy, Foshan Hospital of TCM, with prior compositional analysis of DCHD having confirmed critical quality attributes (Huang et al., 2025a; Yang et al., 2025). DCHD was standardized to a final volume of 200 mL via boiling and concentration, and patients were instructed to orally ingest 100 mL twice daily (morning and evening) under supervision to ensure compliance. Given the lack of established positive control drugs for sepsis and the technical challenges in formulating a placebo for herbal decoction, the placebo group received a 10% DCHD regimen. To ensure blinding integrity, caramel coloring was added to simulate the experimental drug in appearance and taste, maintaining consistency in sensory characteristics (Deng et al., 2025; Zhou et al., 2014).

**TABLE 1:** Detailed information of DCHD.

Chinese name	Latin name	Origin of herb	Medicinal part	Weight (g)	Source
Chaihu	Bupleuri Radix	*Bupleurum chinense* DC.	Root	15	Shanxi, China
Huangqin	Scutellariae Radix	Scutellaria baicalensis Georgi	Root	9	Shandong, China
Dahuang	Rhei Radix et Rhizoma	*Rheum officinale* Baill*.*	Root and rhizome	6	Sichuan, China
Zhishi	Aurantii Fructus Immaturus	*Citrus aurantium* L.	Fruit	9	Hunan, China
Banxia	Pinelliae Rhizoma	*Pinellia ternata* Breit.	Tuber	9	Gansu, China
Baishao	Paeoniae Radix Alba	*Paeonia lactiflora* Pall.	Root	9	Anhui, China
Shengjiang	Zingiberis Rhizoma Recens	*Zingiber officinale* Roscoe.	Rhizome	15	Yunnan, China
Dazao	Jujubae Fructus	*Ziziphus jujuba* Mill.	Fruit	12	Xinjiang, China

### 2.6 Outcome measures

The primary outcomes included: (1) Liver function indicators: ALT, aspartate transaminase (AST), and TBil; (2) Disease severity score: SOFA and Acute Physiology and Chronic Health Evaluation II (APACHE II) scores, and the Minimal Clinically Important Difference (MCID) for the SOFA and APACHE II scores were identified as 2-point and 5-point reduction, respectively (Huang et al., 2025b); (3) 28-day all-cause mortality.

The secondary outcomes included: (1) Infection indicators: C-reactive protein (CRP), procalcitonin (PCT), white blood cell count (WBC) and the percentage of neutrophils (NEU%); (2) Coagulation indicators - INR, Prothrombin Time (PT) and Activated partial thromboplastin time (APTT); (3) Gastrointestinal function indicator: bowel sound; (4) Metabolic and respiratory function indicators: blood lactic acid (LAC) and Oxygenation Index (OI).

### 2.7 Adverse events (AEs)

The safety indicators were any AEs. We closely recorded serious AEs throughout the study, mainly including severe diarrhea, nausea and/or vomiting, and herbal allergy. All AEs were graded for severity (mild, moderate, or severe) according to standard clinical criteria. All participant deaths underwent independent physician review to assess potential causality and attribution to the study drug.

### 2.8 Metabolomics analysis

Blood samples were collected from 6 patients in the DCHD group before and after therapy. Pre-treatment blood was drawn at 8:00 a.m. on the first morning following enrollment, while post-treatment blood was collected 2 h after the final dose administration. After standing for 1 h, samples were centrifuged at 3,000 *g* for 15 min at 4 °C to obtain serum, and then transferred into sterile microcentrifuge tubes and promptly stored at −80 °C for subsequent metabolomic analysis (Li J. et al., 2022).

Serum metabolites were extracted using a methanol/acetonitrile-based method. Briefly, 100 μL of serum was combined with 400 μL of ice-cold extraction solvent (acetonitrile/methanol/water = 2:2:1, v/v), vortex-mixed thoroughly, and incubated on ice for 30 min. Following centrifugation at 14,000 g for 20 min (4 °C), the supernatant was collected and dried in a vacuum centrifuge at 4 °C. For Liquid Chromatography-Tandem Mass Spectrometry (LC-MS/MS) analysis, dried extracts were reconstituted in 100 μL of solvent (acetonitrile/water = 1:1, v/v) and transferred to LC vials. Chromatographic separation was performed on an Agilent 1,290 Infinity Ultra High Performance Liquid Chromatography (UHPLC) system equipped with a Hydrophilic Interaction Liquid Chromatography (HILIC) column. Metabolites were subsequently analyzed by electrospray ionization mass spectrometry (ESI-MS) in both positive and negative ion modes.

Following quality assessment, control, and standardization of the raw data, Principal Component Analysis (PCA) and Partial Least Squares Discriminant Analysis (PLS-DA) models were constructed to discriminate between sample classes and identify significantly altered metabolites. DMs were screened using thresholds of |log_2_ (fold change)| > 1 and *p* < 0.05. Significantly enriched metabolic pathways among DMs were subsequently identified through Over-Representation Analysis (ORA), and False Discovery Rate (FDR) is a statistical metric used to control the proportion of false discoveries in multiple hypothesis testing.

### 2.9 Statistical analysis

Statistical analysis was performed using SPSS 27.0 (IBM, Armonk, New York, United States). For continuous variables with small sample sizes, normality was first assessed using the Shapiro-Wilk test. Data conforming to a normal distribution were expressed as mean ± standard deviation (SD) and analyzed via independent samples t-test: Homogeneity of variance was verified using Levene’s F-test, with the standard t-test applied if variances were equal or Welch’s t′-test if variances were unequal. Paired samples t-test was used for within-group comparisons. Non-normally or partially normally distributed data were reported as median (interquartile range [IQR]; P25, P75) and analyzed using the Mann-Whitney U test for between-group comparisons or the Wilcoxon signed-rank (Z) test for within-group comparisons. Categorical variables were described as frequency and proportion/percentage (n, %), and mean rank ( $\bar{R}$ ), with between-group differences assessed via the Mann-Whitney U test.

Raw data were processed using the XCMS software for peak alignment, retention time correction, and peak area extraction. Metabolite identification was performed by matching experimental data against reference databases based on accurate mass (mass error <25 ppm) and MS/MS spectral matching. Data integrity was subsequently verified, followed by normalization. The processed data were imported into SIMCA-P 16.1 software (Umetrics, Sweden) for multivariate statistical analysis, including PCA and PLS-DA. Univariate statistical analyses comprised Student's t-tests and fold-change analysis. All visualizations were generated using the Metware Cloud Bioinformatics Platform (Wuhan Metware Biotechnology Co., Ltd., China).

## 3 Results

### 3.1 Participants and baseline characteristics

**FIGURE 1:**
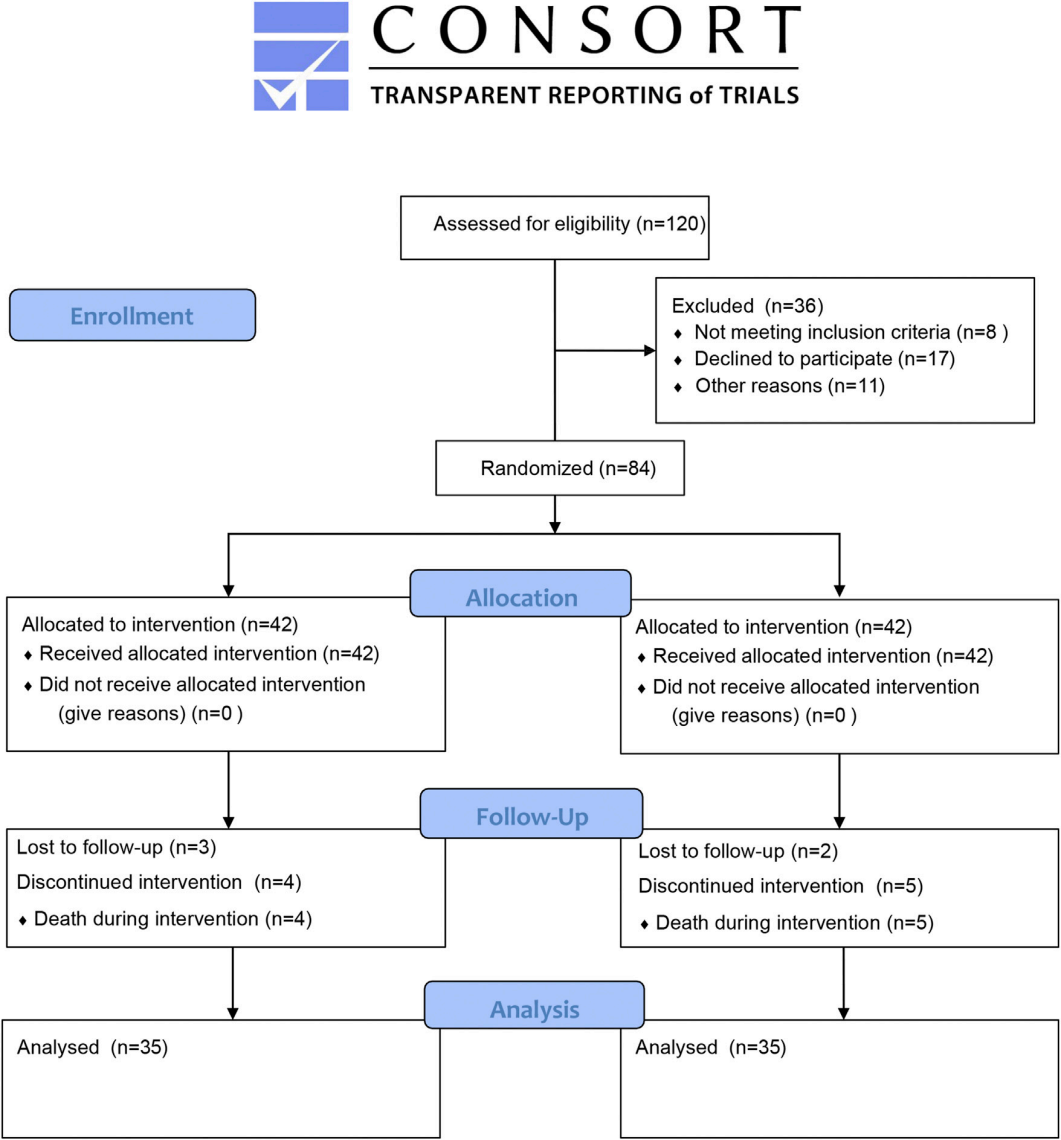
Flow diagram for participant recruitment and follow-up.

In all, 120 patients were initially screened for eligibility in this trial. Following the application of predefined inclusion and exclusion criteria, 36 participants were excluded from the final cohort. 84 eligible patients who met the established clinical parameters for subsequent analysis. During the trial period, 8 patients discontinued participation due to hospital transfers or voluntary discharge (3 cases in the DCHD group, 5 cases in the placebo group). Additionally, 6 patients succumbed to disease progression (4 cases in the DCHD group, 2 cases in the placebo group). The final analysis included 70 participants with balanced allocation (35 per group), completing the predetermined sample size requirement (Figure 1).

There were no statistically significant differences between the two groups regarding the distribution of gender, age, body mass index (BMI), disease duration, personal history, underlying comorbidities, infection sites, pre-treatment SOFA and APACHE II scores, as well as parameters related to organ support therapies and organ functional status. Interestingly, systematic analysis revealed that hypertension, type 2 diabetes, cholelithiasis, and stroke were the most prevalent pre-existing comorbidities in SLI patients. Furthermore, quantitative analysis of infection site across both cohorts identified pulmonary, intra-abdominal, skin and soft tissues, aurinary tract infections as predominant anatomical distributions, and no significant difference in the distribution of infection sites between the two groups. Regarding organ support modalities, invasive mechanical ventilation and continuous renal replacement therapy (CRRT) predominated, whereas artificial liver support systems, extracorporeal membrane oxygenation (ECMO) and intra-aortic balloon pump (IABP) demonstrated significantly lower utilization rates, and also no significant difference in the provision of organ function support therapy between the two groups (Table 2).

**TABLE 2:** Baseline Characteristics of participants.

Variables	Placebo (n = 35)	DCHD (n = 35)	*p* value
Age (year, median [IQR])	67 (51, 77)	57 (49, 70)	0.16
Gender, n (%)			0.78
Female	9 (25.71%)	8 (22.86%)	
Male	26 (74.29%)	27 (77.14%)	
BMI (kg/m^2^, mean ± SD)	24.00 ± 1.76	24.35 ± 1.63	0.397
Disease course (d)	2 (1, 5)	3 (1, 7)	0.398
Smoking, n (%)	22 (62.86%)	24 (68.57%)	0.615
Drinking, n (%)	20 (57.14%)	18 (51.43%)	0.631
Basic illnesses			
Hypertension, n (%)	8 (22.86%)	10 (28.57%)	0.584
Type 2 diabetes, n (%)	6 (17.14%)	7 (20%)	0.759
Cholelithiasis, n (%)	3 (8.57%)	4 (11.43%)	1
Stroke, n (%)	5 (14.29%)	2 (5.71%)	0.428
Chronic obstructive pulmonary disease, n (%)	3 (8.57%)	3 (8.57%)	1
Coronary heart disease, n (%)	2 (5.71%)	4 (11.43%)	0.673
Chronic kidney disease, n (%)	3 (8.57%)	2 (5.71%)	1
Infection site			
Pulmonary	15 (42.86%)	9 (25.71%)	0.131
Intra-abdominal	9 (25.71%)	12 (34.29%)	0.434
Skin and soft tissue	6 (17.14%)	5 (14.29%)	1
Urinary tract	4 (11.43%)	5 (14.29%)	1
Bloodstream	2 (5.71%)	5 (14.29%)	0.428
Central nervous system	1 (2.86%)	1 (2.86%)	1
Qrgan support therapy			
Invasive ventilator	30 (85.71%)	24 (68.57%)	0.088
CRRT	19 (54.29%)	18 (51.43%)	0.811
Artificial liver support systems	2 (5.71%)	1 (2.86%)	1
ECOM	2 (5.71%)	1 (2.86%)	1
IABP	1 (2.86%)	2 (5.71%)	1
Disease severity score			
SOFA (median [IQR])	10.31 ± 4.27	10.37 ± 3.51	0.951
APACHE II (median [IQR])	22 (15, 28)	18 (14, 25)	0.110
Liver function indicators			
ALT (U/L, median [IQR])	45.20 (23.60, 131.80)	59.80 (34.70, 103.00)	0.499
AST (U/L, median [IQR])	88.50 (37.50, 179.20)	77.70 (49.40, 125.50)	0.828
TBil (μmol/l, median [IQR])	47.90 (36.90, 74.40)	52.80 (43.20, 93.20)	0.076
Coagulation indicators			
INR (median [IQR])	1.58 (1.50, 1.80)	1.60 (1.50, 1.80)	0.832
PT (s, median [IQR])	17.2 (16.1, 19.7)	16.3 (14.5, 18.6)	0.053
APTT (s, median [IQR])	37.2 (34.2, 42.7)	36.9 (34.4, 44.8)	0.981
Infection indicators			
WBC (×10^9/L, median [IQR])	14.92 (10.71, 19.55)	11.92 (10.03, 17.50)	0.169
NEU% (median [IQR])	88.6 (84.1, 93.2)	85.9 (80.5, 91.2)	0.188
PCT (ng/ml, median [IQR])	8.27 (2.33, 19.14)	6.97 (1.45, 10.33)	0.267
CRP (mg/L, median [IQR])	124.26 (63.20, 142.43)	122.43 (47.15, 158.39)	0.720
Gastrointestinal function indicator			
Bowel sound	2 (2, 3)	1 (2, 3)	0.422
Metabolic and respiratory function indicators			
LAC (mmol/L, median [IQR])	1.90 (1.40, 2.75)	1.90 (1.20, 3.30)	0.930
OI (mmHg, mean ± SD)	302.46 ± 97.71	311.97 ± 122.41	0.721

SD, standard deviation; IQR, interquartile range; BMI, body mass index; CRRT, continuous renal replacement therapy; ECMO, extracorporeal membrane oxygenation; IABP, intra-aortic balloon pump; SOFA, Sequential Organ Failure Assessment; APACHE II, Acute Physiology and Chronic Health Evaluation II; ALT, alanine transaminase; AST, aspartate transaminase; TBil, total bilirubin; INR, International normalized ratio; PT, Prothrombin Time; APTT, Activated partial thromboplastin time; CRP, C-reactive protein; PCT, procalcitonin; WBC, white blood cell; NEU, neutrophil; LAC, lactic acid; OI, oxygenation index.

Of particular note, descriptive statistics were used to analyze the distribution patterns of infectious pathogens between the two cohorts revealed Gram-negative bacilli predominance, with *Klebsiella pneumoniae*, *Escherichia coli*, *Pseudomonas aeruginosa*, and *Acinetobacter baumannii* constituting the predominant isolates. Gram-positive pathogens were typified by *Staphylococcus aureus*, while fungal spectra featured *Aspergillus spp*. and *Candida albicans* (Table 3).

**TABLE 3:** Pathogen distribution.

Pathogen	Placebo (n = 35)	Note	DCHD (n = 35)	Note
*Klebsiella pneumoniae*	6		6	
*Escherichia coli*	6		5	
*Pseudomonas aeruginosa*	6		4	
*Acinetobacter baumannii*	6	1 Extensively drug-resistant	3	1 Multidrug-resistant
*Staphylococcus aureus*	4		2	1 MRSA
*Enterococcus faecium*	0		3	
*Aspergillus spp.*	0		3	
*Candida albicans*	1		1	
*Candida tropicalis*	1		1	
*Edwardsiella tarda*	1		0	
*Streptococcus agalactiae*	1		0	
*Streptococcus pneumoniae*	1		0	
*Enterobacter cloacae*	1		0	
*Enterococcus faecalis*	1		0	
*Enterobacter cloacae complex*	1		0	
*Staphylococcus haemolyticus*	0		1	
*Chryseobacterium indologenes*	0		1	1 Multidrug-resistant
*Candida albicans*	0		1	
*Cryptococcus spp.*	0		1	
*Staphylococcus epidermidis*	0		1	
*Serratia marcescens*	0		1	
**SARS-CoV-2**	0		1	

MRSA, methicillin-resistant Staphylococcus aureus; SARS-CoV-2, severe acute respiratory syndrome coronavirus 2.

### 3.2 Primary outcomes

A systematic summary of the primary outcomes was provides in Table 4. (1) Liver function indicators: Compared with baseline levels, ALT and AST levels decreased significantly in the placebo group after 5-day treatment (31.50 (IQR, 19.60, 64.70) *vs.* 45.20 (IQR, 23.60, 131.80), *p* = 0.046 for ALT, and 45.30 (IQR, 24.20, 110.30) *vs.* 88.50 (IQR, 37.50, 179.20), *p* = 0.005 for AST), with the DCHD treatment group exhibiting a significantly greater decline (32.80 (IQR, 19.90, 50.70) *vs.* 59.80 (IQR, 34.70, 103.00), *p* < 0.001 for ALT, and 35.00 (IQR, 24.00, 68.50) *vs.* 77.70 (IQR, 49.40, 125.50), *p* < 0.001 for AST). However, no statistically significant differences were observed between the two groups in either post-treatment ALT and AST levels or their changes from baseline (*p >* 0.05). Remarkably, compared to baseline, the DCHD treatment group exhibited a statistically significant difference in TBil after 5-day treatment (52.80 (IQR, 43.20, 93.20) *vs.* 36.30 (IQR, 22.80, 62.70), *p* < 0.001), and TBil changes from baseline to post-treatment in the DCHD group were markedly lower compared to the placebo group (−22.50 (IQR, −37.20, −8.10) *vs.* −3.30 (IQR, −17.16, 12.40), *p* < 0.001). (2)Disease severity scores: Compared to baseline, the placebo group exhibited significant reductions in both SOFA ((9.20 ± 5.18) *vs.* (10.31 ± 4.27), *p =* 0.02) and APACHE II (22 (IQR, 15, 28) *vs.* 22 (IQR, 15, 28), *p =* 0.024) scores post-treatment, while the DCHD treatment group demonstrated more pronounced declines ((7.91 ± 4.72) *vs.* (10.37 ± 3.51), *p* < 0.001for SOFA, and 16 (IQR, 10, 21) *vs.* 18 (IQR, 14, 25), *p* < 0.001 for APACHE II). Compared to the placebo group, no statistically significant difference was observed in SOFA score in the DCHD group after 5-day treatment ((7.91 ± 4.72) vs. (9.20 ± 5.18), *p* = 0.951), whereas APACHE II score showed significant intergroup variation (16 (IQR, 10, 21) *vs.* 22 (IQR, 15, 28), *p =* 0.011). Notably, the DCHD group displayed statistically significant mean differences in both SOFA ((-2.46 ± 2.84) *vs.* (−1.11 ± 2.71), *p* = 0.047) and APACHE II (−5 (IQR, −5, −2) *vs.* −2 (IQR, −5, 2), *p* = 0.034) scores when comparing baseline to post-treatment changes. Surprisingly, SOFA and APACHE II scores in the DCHD group decreased beyond MCID thresholds. (3) The DCHD group and the placebo group showed no significant difference in 28-day all-cause mortality (7 (20.0%) *vs.* 9 (25.7%), *p =* 0.569). These preliminary findings demonstrated that DCHD intervention exhibited clinically meaningful hepatoprotective effects in SLI patients, as evidenced by significant improvement in hepatic functional parameters, particularly in TBil. Furthermore, DCHD treatment was associated with marked amelioration of disease severity, manifested by statistically significant reductions in both SOFA and APACHE II scores.

**TABLE 4:** Primary outcomes.

Primary outcomes	The fifth day			Change from baseline		
	Placebo (n = 35)	DCHD (n = 35)	*p* value	Placebo (n = 35)	DCHD (n = 35)	*p* value
ALT (U/L, median[IQR])	31.50 (19.60, 64.70)^#^	32.80 (19.90, 50.70)^###^	0.756	-8.90 (-42.50, 4.10)	-21.60 (-48.50, -4.00)	0.288
AST (U/L, median[IQR])	45.30 (24.20, 110.30)^##^	35.00 (24.00, 68.50)^###^	0.388	-14.50 (-97.50, 3.60)	-33.00 (-66.40 ,-8.00)	0.553
TBil (μmol/l, median[IQR])	45.60 (27.90, 90.20)	36.30 (22.80, 62.70)^###^	0.307	-3.30 (-17.16, 12.40)	-22.50 (-37.20, -8.10)^***^	< 0.001
SOFA (median[IQR])	9.20 ± 5.18^#^	7.91 ± 4.72^###^	0.281	-1.11 ± 2.71	-2.46 ± 2.84^*^	0.047
APACHE II (median[IQR])	22 (15, 28)^#^	16 (10, 21)^###*^	0.011	-2 (-5, 2)	-5 (-5, -2)^*^	0.034

Compared with before treatment, ^#^ *p* < 0.05, ^##^ *p* < 0.01, ^###^ *p* < 0.001; Comparison with placebo group, ^*^ *p* < 0.05, ^***^ *p* < 0.001. SD, standard deviation; IQR, interquartile range; ALT, alanine transaminase; AST, aspartate transaminase; TBil, total bilirubin; SOFA, Sequential Organ Failure Assessment; APACHE II, Acute Physiology and Chronic Health Evaluation II.

### 3.3 Secondary outcomes

Secondary outcomes were presented in Table 5. (1) Infection indicators: Compared with baseline levels, WBC, NEU%, PCT, and CRP decreased significantly in the placebo group after 5-day treatment (10.50 (IQR, 8.73, 16.53) *vs.*14.92 (IQR, 10.71, 19.55), *p =* 0.004 for WBC, 82 (IQR, 76.2, 88.4) *vs.* 88.6 (IQR, 84.1, 93.2), *p* < 0.001 for NEU%, 1.50 (IQR, 0.85, 6.01) *vs.* 8.27 (2.33, 19.14), *p* < 0.001 for PCT, and 68.29 (IQR, 30.7, 125.68) *vs.* 124.26 (IQR, 63.20, 142.43) *p* = 0.004 for CRP). Meanwhile, the DCHD group also exhibited decreased levels, with a significantly greater reduction in WBC and CRP levels as well (9.70 (IQR, 7.60, 10.98) *vs.* 11.92 (IQR, 10.03, 17.50), *p* < 0.001 for WBC, 76.6 (IQR, 70.8, 86.3) *vs*. 85.9 (IQR, 80.5, 91.2), *p* < 0.001 for NEU%, 1.08 (IQR, 0.28, 2.73) *vs*. 6.97 (1.45, 10.33), *p* < 0.001 for PCT, and 70.00 (IQR, 18.4, 132.74) *vs*. 122.43 (IQR, 47.15, 158.39), *p* < 0.001 for CRP. Remarkably, compared with the placebo group, only PCT levels differed significantly in the DCHD group after treatment (1.08 (IQR, 0.28, 2.73) *vs*. 1.50 (IQR, 0.85, 6.01), *p =* 0.030). Moreover, no statistically significant differences were observed between the two groups in the changes from baseline for WBC, NEU%, PCT, or CRP levels (*p >* 0.05). (2) Coagulation indicators: Compared with baseline levels, the placebo group exhibited a significant reduction in INR and PT, while a non-significant decrease in APTT after 5 days of treatment (1.30 (IQR, 1.14, 1.46) *vs.* 1.58 (IQR, 1.50, 1.80), *p =* 0.001 for INR, 14.1 (IQR, 12.6, 16.2) *vs*. 17.2 (16.1, 19.7), *p* = 0.003 for PT, 36.8 (IQR, 32.3, 41.5) *vs*. 37.2 (34.2, 42.7) for APTT, *p* = 0.122). Meanwhile, the DCHD group exhibited more pronounced reductions in both INR, PT and APTT after 5 days of treatment (1.20 (IQR, 1.10, 1.40) *vs.* 1.60 (IQR, 1.50, 1.80), *p* < 0.001 for INR, 13.4 (IQR, 12.4, 14.4) *vs*. 16.3 (IQR, 14.5, 18.6), *p* < 0.001 for PT, 35 (IQR, 32.3, 41.9) *vs*. 36.9 (IQR, 34.4, 44.8), *p =* 0.004 for APTT). However, no significant differences were observed between the two groups in the changes from baseline for INR, PT and APTT (*p >* 0.05). (3) Gastrointestinal function indicator: Compared with baseline levels, the placebo group exhibited a significant increase in bowel sounds (3 (IQR, 2, 4) *vs*. 2 (IQR, 2, 3), *p =* 0.013),while the DCHD group manifested a more pronounced elevation (3 (IQR, 2, 4) *vs*. 1 (IQR, 2, 3), *p* < 0.001). Nevertheless, compared with the placebo group, the DCHD treatment group showed no significant differences in either post-treatment bowel sounds or the changes from baseline (*p >* 0.05). (4) Metabolic and respiratory function indicators: Compared with baseline levels, a significant decrease in LAC was observed in the placebo group after 5 days of treatment (1.40 (IQR, 0.90, 2.38) *vs*. 1.90 (IQR, 1.40, 2.75), *p* = 0.041), while no significant difference was found in OI (*p >* 0.05). By comparison, LAC and OI decreased significantly in the DCHD group after 5-day treatment (1.20 (IQR, 0.70, 2.00) *vs*. 1.90 (IQR, 1.20, 3.30), *p* = 0.009 for LAC, 341.68 ± 99.45 *vs*. 311.97 ± 122.41, *p =* 0.025 for OI). Notably, compared with the placebo group, the DCHD group demonstrated no significant differences in post-treatment LAC levels or the changes from baseline (*p >* 0.05). However, significant differences were observed in both post-treatment OI (341.68 ± 99.45 *vs*. 287.30 ± 106.95, *p* = 0.031) and the changes from baseline (29.71 ± 74.76 *vs*. −15.16 ± 108.51, *p* = 0.048) following DCHD treatment. Collectively, DCHD demonstrates efficacy in ameliorating systemic infection, coagulation function, gastrointestinal function, metabolic status, and respiratory function in patients with SLI to a certain extent, with particularly significant efficacy in reducing PCT levels and improving OI.

**TABLE 5:** Second outcomes.

Second outcomes	The fifth day			Change from baseline		
	Placebo (n = 35)	DCHD (n = 35)	*p* value	Placebo (n = 35)	DCHD (n = 35)	*p* value
Infection indicators						
WBC (×10^9/L, median[IQR])	10.50 (8.73, 16.53)^##^	9.70 (7.60, 10.98)^###^	0.062	-2.25 (-6.83, -0.05)	-2.48 (-7.37, -0.69)	0.438
NEU% (median[IQR])	82 (76.2, 88.4)^###^	76.6 (70.8, 86.3)^###^	0.082	-5.7 (-8.6, -0.3)	-6.1 (-12.4, -1.6)	0.823
PCT (ng/ml, median[IQR])	1.50 (0.85, 6.01)^###^	1.08 (0.28, 2.73)^*###^	0.030	-3.91 (-10.26, -1.04)	-4.63 (-9.31, -0.47)	0.783
CRP (mg/L, median[IQR])	68.29 (30.7, 125.68)^##^	70.00 (18.4, 132.74)^###^	0.792	-25.49 (-59.75, 5.42)	-27.77 (-69.30, -8.69)	0.685
Coagulation indicators						
INR (median[IQR])	1.30 (1.14, 1.46)^##^	1.20 (1.10, 1.40)^###^	0.127	-0.30 (-0.50, -0.10)	-0.40 (-0.50, -0.20)	0.065
PT (s, median[IQR])	14.1 (12.6, 16.2)^##^	13.4 (12.4, 14.4)^###^	0.121	-3.5 (-5.2, -0.5)	-2.5 (-5.1, -1.1)	0.833
APTT (s, median[IQR])	36.8 (32.3, 41.5)	35 (32.3, 41.9)^##^	0.725	-1.5 (-8.4, 3.1)	-2.3 (-6.4, -0.2)	0.537
Gastrointestinal function indicator						
Bowel sound	3 (2, 4)^#^	3 (2, 4)^###^	0.961	0 (-1, 2)	1 (0, 2)	0.459
Metabolic and respiratory function indicators						
LAC (mmol/L, median[IQR])	1.40 (0.90, 2.38)^#^	1.20 (0.70, 2.00)^##^	0.215	-0.50 (-1.30, 0.26)	-0.60 (-1.77, 0.70)	0.417
OI (mmHg, mean ± SD)	287.30 ± 106.95	341.68 ± 99.45^#*^	0.031	-15.16 ± 108.51	29.71 ± 74.76^*^	0.048

Compared with before treatment, ^#^ *p* < 0.05, ^##^ *p* < 0.01, ^###^ *p* < 0.001; Comparison with placebo group, ^*^ *p* < 0.05. SD, standard deviation; IQR, interquartile range; CRP, C-reactive protein; PCT, procalcitonin; WBC, white blood cell; NEU, neutrophil; INR, International normalized ratio; PT, Prothrombin Time; APTT, Activated partial thromboplastin time; LAC, lactic acid; OI, oxygenation index.

### 3.4 Safety

Six patients died during the study, including 4 in the DCHD group and 2 in the placebo group, but they were judged not related to the study drug. Nonfatal AEs occurred in 4 patients (11.43%) in the DCHD group and 2 (5.71%) in the placebo group (*p* = 0.673), mainly driven by gastrointestinal symptoms such as abdominal distension (1 patient in the placebo group), diarrhea (1 patient in the placebo group and 2 patients in the DCHD group) and vomiting (1 patient in the DCHD group). and rash attributable to herbal medicine hypersensitivity (1 patient in the DCHD group). Add: All these events were mild in severity and were assessed as being closely associated with the patients’ underlying conditions. No participant experienced serious AEs.

### 3.5 Results of metabolomics analysis

**FIGURE 2:**
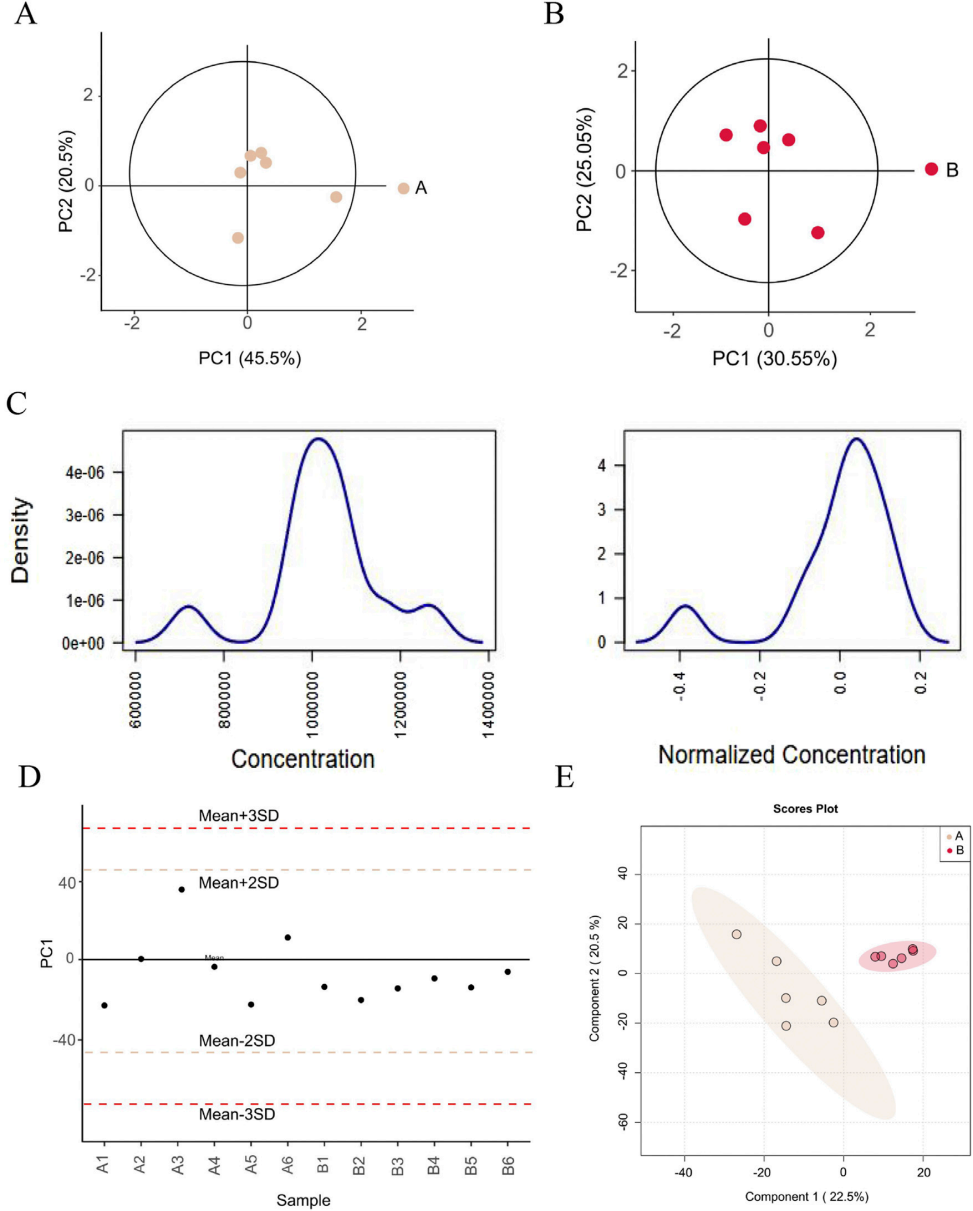
Metabolomics analysis. **(A)** PCA score diagram of pre-treatment DCHD group in positive and negative ion modes. **(B)** PCA score diagram of post-treatment DCHD group in positive and negative ion modes. **(C)** Normalization of samples. **(D)** Distribution of PC1 scores across sample cohorts. Sample identifiers are displayed on the horizontal axis. Sample identifiers are displayed on the horizontal axis, with the vertical axis indicating respective PC1 loadings from PCA. **(E)** PLS-DA score plot. Each point in the figure corresponds to a sample, and the horizontal and vertical coordinates are the values of the two factors with the best discriminant effect. Different groups are marked by different colors, and the area indicated by the ellipse is the 95% confidence region of the sample points.

PCA results demonstrate compact clustering of samples within the 95% confidence ellipse, indicating the overall metabolic background of the subjects remained relatively stable (Figures 2A,B). Prior to standardization, metabolite concentrations exhibited marked heterogeneity, reflected in disparate median values and IQRs across samples. Following standardization, these values were effectively aligned to a uniform baseline (Figure 2C). Furthermore, the distribution of PC1 scores revealed that the majority of samples varied within 2 SDs of the mean axis, signifying that all samples remained within acceptable experimental variation (Figure 2D). PLS-DA analysis was employed to evaluate inter-group structural differences in metabolite composition. Results demonstrated effective class separation between the two groups under both positive and negative ionization modes, indicating robust discriminatory power of the PLS-DA model and the presence of significantly DMs (Figure 2E). 92 significant DMs across both positive and negative ionization modes were identified, comprising 37 downregulated and 55 upregulated DMs. The detailed information of DMs is presented in Table 6. These DMs were predominantly represented by flavonoids (Wogonin, Wogonoside, Tectoridin), bile acids (Stearidonic acid, Cholic acid, and Glycocholic acid), amino acid derivatives (Djenkolic Acid, His-Pro, DL-phenylalanine, and Glutamic acid), and alkaloids (Betonicine and Stachydrine). Table 7 shows the top 7 metabolic pathways of DMs, including Alanine, aspartate and glutamate metabolism, Bile acid metabolism, Degradation of flavonoids, Glutathione metabolism, Pyrimidine metabolism, Amino sugar and nucleotide sugar metabolism, and Phenylalanine metabolism. Of particular interest, the bile acid metabolism pathway demonstrated a discernible enrichment trend (p < 0.05); however, it did not surpass the predetermined statistical threshold for false discovery rate (FDR = 0.094). This nominally significant result suggests that bile acid metabolism may represent a putative target through which DCHD ameliorates SLI, warranting further validation in subsequent studies employing more precise analytical techniques.

**TABLE 6:** Differential metabolites.

No.	Metabolite	RT (s)	KEGG ID	m/z	Adduct	log₂FC	*P* value
1	Betonicine	160.09577	C08269	289.772	[M+H]+	4.2486	5.6065e-07
2	Djenkolic Acid	253.05538	C08275	32.226	[M-H]-	3.7151	1.2746e-05
3	Wogonin	285.07567	C10197	40.098	[M+H]+	4.7843	1.5822e-05
4	Stearidonic acid	277.21497	C16300	194.791	[M+H]+	-4.4466	0.00980
5	Wogonoside	461.10685	NA	197.590	[M+H]+	2.8389	0.00163
6	Cholic acid	355.26169	C00695	220.560	[M+H-3H2O]+	-2.2835	0.01004
7	Glycocholic acid	464.32122	C01921	182.523	[M-H]-	-3.4090	0.01146
8	Tectoridin	301.06987	C10533	36.279	[M+H-C6H10O5]+	4.2974	0.00076
9	His-Pro	235.11765	NA	146.182	[M+H-H2O]+	1.7077	0.00052
10	DL-phenylalanine	166.08523	C02057	255.934	[M+H]+	-2.3794	0.02199
11	Stachydrine	144.10083	C10172	271.154	[M+H]+	3.4566	3.5094e-05
12	Glutamic acid	148.05936	C00025	391.553	[M+H]+	-1.5843	0.02815

**TABLE 7:** Metabolic pathways.

Metabolic pathway	Total	Hits	-log p	Impact	FDR
Alanine, aspartate and glutamate metabolism	60	4	3.380	0.167	0.069
Bile acid metabolism	35	3	2.941	0.033	0.094
Degradation of flavonoids	11	2	2.726	0.6	0.103
Glutathione metabolism	62	3	2.227	0.065	0.245
Pyrimidine metabolism	68	3	2.115	0.014	0.253
Amino sugar and nucleotide sugar metabolism	28	2	1.916	0.026	0.333
Phenylalanine metabolism	35	2	1.731	0.101	0.438

## 4 Discussion

Current evidence regarding effective and safe therapies for SLI remains limited. The classic Chinese herbal formula DCHD, known for its actions in soothing liver-gallbladder (*shu gan li dan*), and dredging the fu organs and draining heat (*tong fu xie re*), is extensively utilized in clinical practice for SLI management, yet it lacks credible clinical validation. This prospective, single-center, single-blind randomized controlled trial provides the first clinical evidence for the efficacy and safety of DCHD in treating SLI. In this randomized clinical trial, as an adjunctive TCM therapy integrated with guideline-directed treatment, DCHD demonstrated significant improvements in liver function, disease severity, systemic inflammation, coagulation parameters, gastrointestinal injury, and metabolic and respiratory functions in SLI patients. Our study delivers pivotal evidence for the clinical decision-making for DCHD in SLI patients.

Diagnosis and treatment of SLI remain a great clinical challenge in infectious diseases for clinicians. Commonly used liver assessment tools like the Child-Pugh score, Model for End-stage Liver Disease, and Albumin-Bilirubin score were primarily developed for chronic liver disease or non-infectious liver failure. Consequently, specific diagnostic criteria for SLI are still lacking. Notably, these scoring systems typically incorporate the INR, a measure of coagulation function. To our knowledge, liver injury often stems from ischemia, cytokine storm, or direct hepatocyte damage by endotoxins during the pathogenesis of sepsis, leading to reduced synthesis of coagulation factors and elevated INR (Mawatari et al., 2018). Therefore, the INR serves as an indirect marker of reduced hepatic synthetic capacity, reflecting the degree of hepatocyte dysfunction. It is important to note that INR values can be influenced by anticoagulant medications, which may potentially confound the diagnosis of SLI(Liew et al., 2013). SOFA score, a standard tool for evaluating organ dysfunction and aiding sepsis diagnosis, defines hepatic dysfunction as a TBil level ≥2 mg/dL (Ferreira et al., 2001). ALT is predominantly located in the hepatocyte cytoplasm, exhibits high specificity for the liver, and an elevation in ALT typically signifies direct hepatocellular damage. In contrast, AST is widely distributed in tissues including the liver, myocardium, skeletal muscle, and kidneys, resulting in lower specificity. Therefore, an elevated AST level in sepsis may originate from multi-organ injury rather than solely indicating hepatic damage (Li et al., 2019). Several studies define SLI using the criteria of TBil >2 mg/dL and INR >1.5, applied in patients meeting the diagnosis of sepsis (He et al., 2024; Wen et al., 2024; Xie et al., 2022). In contrast, a study has also proposed a diagnostic standard for SLI comprising TBil >34.2 μmol/L and INR >1.5 or ALT exceeding twice the upper limit of normal (Song et al., 2025). Therefore, utilizing a combination of TBil, INR, and ALT as diagnostic parameters provides a more comprehensive assessment of SLI.

Multiple studies have confirmed that individuals with underlying conditions such as diabetes and hypertension are at increased risk of developing sepsis, with specific diseases like pneumonia and cholecystitis serving as significant predisposing factors (Cavallazzi et al., 2020; Kuo et al., 1995). Among the seventy patients enrolled in this study, metabolic disorders (e.g., diabetes), cardiovascular/cerebrovascular diseases (e.g., hypertension, coronary artery disease, stroke), and chronic organ dysfunction (e.g., chronic obstructive pulmonary disease, chronic renal failure) were predominant comorbidities. However, the potential association between these underlying conditions and the development of SLI requires further investigation. Epidemiological evidence indicates that pulmonary, hematogenous, and intra-abdominal infections represent the primary sources of sepsis (Xie et al., 2020). Our findings align with previous reports, identifying pulmonary, abdominal, and urinary tract infections as the most common infection sources. Notably, intra-abdominal infection may be the predominant site of infection associated with SLI, and abdominal pathologies such as acute perforated appendicitis and cholangitis are significant risk factors for liver injury, with intra-abdominal infections being significantly more prevalent in the SLI group (34.1%) compared to the non-liver injury group (21.7%) (Sun, 2019). The underlying mechanism involves disruption of the intestinal barrier, which facilitates bacterial and endotoxin translocation via the gut-liver axis, thereby contributing to the initiation or exacerbation of liver damage. This underscores the critical role of the gut-liver axis in the pathogenesis of SLI (Zhang et al., 2022). Retrospective microbiological analysis revealed that Gram-negative bacteria predominated (60%) among pathogens identified in 138 SLI patients, with *Escherichia coli* being the most prevalent. *Escherichia coli* and *Acinetobacter baumannii* were the primary Gram-negative isolates from abdominal and pulmonary sources, respectively, while *Enterococcus faecium* and *Candida albicans* were the most common Gram-positive bacterium and fungus (Sun, 2019). Consistent with prior research, our study of 70 patients also demonstrated an absolute predominance of Gram-negative bacteria (e.g., *Klebsiella pneumoniae*, *Escherichia coli*, *Pseudomonas aeruginosa*, *A. baumannii*), Gram-positive bacteria and fungi were less frequently isolated, represented by *Staphylococcus aureus* and *Aspergillus spp*., respectively.

Organ support technologies play a pivotal role in the management of SLI, and invasive ventilator, CRRT, ECMO, and IABP are currently common extracorporeal life support techniques employed in the ICU to sustain vital organ functions. Septic patients frequently present with acute respiratory distress syndrome (ARDS), and mechanical ventilation support can improve oxygenation by adjusting respiratory parameters, thereby mitigating hypoxemia-induced hepatocellular injury (Kim et al., 2024). Early initiation of CRRT may positively impact the prognosis of septic patients, because it can effectively clear toxins, regulate fluid and electrolyte balance, and remove inflammatory mediators (An et al., 2023). While the feasibility of ECMO for adults with sepsis-induced respiratory and circulatory failure remains controversial, it may attenuate liver injury by improving oxygenation and reducing the systemic inflammatory response (Mai et al., 2025). Our findings indicate that invasive ventilation and CRRT were used more frequently among the 70 patients, whereas artificial liver support systems, ECMO, and IABP were applied relatively less. This pattern suggests that SLI patients often develop multiple organ dysfunction syndrome (MODS), necessitating invasive ventilation for respiratory support and CRRT for toxin clearance and homeostasis. The comparatively lower utilization of ECMO and artificial liver support may also be attributable to their high costs and technical complexity.

Liver function, SOFA and APACHE II scores, and 28-day all-cause mortality served as the primary efficacy endpoints in this study, because DCHHD can effectively improve systemic inflammation symptoms by soothing liver-gallbladder (*shu gan li dan*) and clearing heat and clearing heat-toxin (*qing re jie du*), thus exerting hepatoprotective effect (Liver function) and reducing disease severity (SOFA and APACHE II scores). For critically ill patients, the paramount objective of any therapeutic intervention is to reduce mortality and improve survival. The 28-day all-cause mortality rate represents a hard endpoint and the gold standard for evaluating the efficacy of critical care interventions. Our results indicate that DCHD can improve liver function (especially TBil), SOFA and APACHE II scores in patients with SLI. AST and ALT are widely used clinical biochemical markers of hepatocellular injury, with elevated levels typically reflecting the degree of hepatocyte damage (Li et al., 2019). Our study revealed comparable levels of ALT and AST between the two groups after treatment, a finding potentially attributable to insufficient sample size and baseline imbalances, which may warrant further subgroup analysis. Conversely, TBil serves as a key indicator for diagnosing and quantifying jaundice, suggesting underlying hepatic injury or biliary obstruction. Ischemic and hypoxic hepatitis caused by ischemia and shock, and cholestatic liver dysfunction resulting from impaired bile metabolism are common clinical types of SLI(Woznica et al., 2018). The decrease in TBil provides preliminary evidence that DCHD effectively enhances the ability of hepatocytes to process bilirubin and promotes biliary excretion. Liver injury in sepsis often involves complex pathological mechanisms, including cytokine storms (e.g., TNF-α, IL-6), microcirculatory disturbances, endotoxin assault, and cholestasis (Wang et al., 2014). As a classical compound formula, Our previous research found that the core compounds of DCHD include Baicalin, Nobiletin, Emodin, and Paeoniflorin, ect (Huang et al., 2025a). Research has demonstrated that baicalin ameliorates non-alcoholic fatty liver disease, ulcerative colitis, cholestasis, and liver fibrosis by modulating upstream oxidative stress and inflammation to participate in downstream pathways of cell death and immune responses. Furthermore, baicalin regulates gut microbiota through promoting the production of short-chain fatty acids, thereby participating in gut-liver axis interactions (Hu et al., 2021). In addition, Recent research has found that in α-naphthylisothiocyanate -induced cholestatic rats, Emodin may exert hepatoprotective effects by modulating bile acid metabolism and reducing hepatic bile acid load, potentially through Sirtuin 1-mediated activation of the farnesoid X receptor (Hu et al., 2024). Interestingly, related research have also revealed that Paeoniflorin can be used to treat cholestatic liver injury in rats induced by bile duct ligation by modulating key transporters in the bile acid signaling pathway of enterohepatic circulation (Wei et al., 2020). This multi-target synergistic mechanism, especially through the regulation of the farnesoid X receptor pathway, anti-inflammatory pathways (TLR4/NF-κB, NLRP3), and the gut-liver axis, likely underlies the advantage of DCHD alleviates SLI by ameliorating bile acid metabolism. Importantly, the effect of DCHD on reducing INR levels in patients was not significant.

Compared to liver function tests, the SOFA and APACHE II scores hold greater clinical value for assessing disease severity, predicting prognosis, and guiding treatment decisions in septic patients. SOFA score is often preferred for initial and rapid assessment due to its relative simplicity and ease of calculation. Conversely, APACHE II score is generally more suitable for long-term monitoring and prognostic evaluation once the patient’s condition has stabilized. Importantly, APACHE II score demonstrates superior predictive accuracy for 28-day mortality in septic patients compared to SOFA score (Knaus et al., 1985; Vincent et al., 1996). The MCID has become increasingly pivotal in clinical trials for evaluating therapeutic efficacy. In this study, we employed the MCID to quantify the impact of DCHD on SOFA and APACHE II scores in septic patients. Although the DCHD intervention failed to improve 28-day mortality due to its limited duration, our findings revealed that both SOFA and APACHE II scores exhibited measurable declines following 5-day of DCHD administration. Critically, the reduction in SOFA and APACHE II scores within the DCHD cohort surpassed the established MCID threshold of 2-point and 5-point. This indicates that DCHD treatment may be beneficial for septic patients and improve prognosis. Besides, previous research revealed that a 3-day course of Wenyang Baidu Yin treatment significantly decreased SOFA and APACHE II scores among septic patients (APACHE II score: 15.0 (IQR, 12.2, 16.0) *vs.* 17.0 (IQR, 13.5, 20.0), SOFA score: 6.0 (IQR, 6.0, 8.0) *vs.* 10.0 (IQR, 8.0, 13.0), *p* < 0.05) (Liang et al., 2024). Intriguingly, a multicenter randomized controlled trial indicated that APACHE II scores (acute gastrointestinal injury (AGI) Grade II: 13.75 ± 3.31 *vs.* 15.37 ± 3.35, *p* = 0.013, AGI Grade III: 12.55 ± 3.56 *vs.* 14.22 ± 3.44, *p* = 0.016, AGI Grade IV: 14.25 ± 4.03 *vs.* 19.25 ± 4.65, *p* = 0.01) in the intervention group were significantly reduced after patients received an initial 7-day course of proper TCM decoction (pills of fructus cannabis, Dachengqi decoction, Chaihushugan powder, or Buzhongyiqi decoction) based on their syndrome differentiation, followed by TCM syndrome type reassessment and a subsequent 7-day course of treatment (Xing et al., 2019). As known, sepsis progresses rapidly. From the perspective of TCM theory, syndrome types demonstrate dynamic evolution, and TCM interventions are typically administered in short courses of 3–7 days to allow for timely therapeutic adjustments based on TCM syndrome type shifts. Therefore, in this study, septic patients exhibiting appropriate TCM syndrome types received DCHD treatment for a 5-day intervention period, while those presenting with deficiency syndrome types were excluded.

DCHD can also reduce the levels of infection-related markers in SLI patients to a certain extent, especially PCT levels, thereby mitigating systemic inflammatory responses. It is well known that cytokine storm is a core pathological mechanism underlying sepsis development. The elevated levels of WBC and NEU% in septic patients are important manifestations of the inflammatory response in the body, indicating the presence of infection and closely related to the severity of the disease, but their sensitivity and specificity are insufficient (Gai et al., 2018). In contrast, PCT plays a pivotal role in the diagnosis and prognosis assessment of sepsis due to its high sensitivity and specificity. PCT levels correlate positively with sepsis severity, are markedly elevated in patients with severe sepsis or septic shock, and their dynamic changes reflect disease progression (Li R. et al., 2022). As a cardinal biomarker of acute inflammatory responses, CRP demonstrates substantial elevation in septic patients, exhibiting a strong correlation with disease severity (Li and Gong, 2018). Consequently, monitoring its longitudinal trajectories provides a clinically actionable metric to gauge therapeutic response and infection control. The previous study of our research group confirmed that DCHD significantly could reduce serum inflammatory factor levels (e.g., IL-1β, TNF-α, IL-6) in rats with sepsis induced by cecal ligation and puncture (Huang et al., 2023). Moreover, extensive basic studies have also shown that DCHD can effectively inhibit the secretion of pro-inflammatory mediators, attenuate tissue inflammatory infiltration, and ameliorate liver injury induced by cholestasis as well as acute pancreatitis (Zhao et al., 2019; Zhou et al., 2022). The above suggests that DCHD may improve the inflammatory state of the body by regulating the inflammatory immune response.

MODS represents the most critical and life-threatening pathophysiological manifestation in the progression of sepsis, epitomizing the ultimate systemic consequences of dysregulated inflammatory responses and immune homeostasis breakdown that inflict widespread tissue damage (Singer et al., 2016). The intestine is considered as the “motor” of MODS in sepsis, indicating the prevalent gastrointestinal dysfunction in septic patients, and characterized by abdominal distension, constipation, and diminished bowel sounds (Payen, 2020). From the perspective of TCM theory, gastrointestinal dysfunction in sepsis primarily stems from bowel-qi obstruction due to endotoxin accumulation and pathogen invasion coupled with stagnation of qi dynamics, and DCHD ameliorates gastrointestinal function by clearing heat and promoting bowel motility. Our findings indicate that both groups exhibited significant improvement in bowel sounds following treatment. However, given the inherent subjectivity and low sensitivity of bowel sound assessment, additional serum biomarkers—such as intestinal fatty acid-binding protein and D-lactate may be employed as supplementary evidence to comprehensively evaluate the impact of DCHD on gastrointestinal function in sepsis patients. Our previous research also showed that DCHD can improve the α and β diversity of intestinal flora in rats, increasing the abundance of beneficial bacteria and reducing the abundance of pathogenic bacteria in CLP-induced rat model of sepsis, which is also an important reason for improving gastrointestinal function in septic patients (Huang et al., 2023). Importantly, Our study demonstrated DCHD can improve OI in septic patients (*p* < 0.05). According to the theory of TCM, the lung and the large intestine have an exterior-interiorly relationship. Therefore, the potential explanation for the result was that DCHD could purge the intestines and remove the toxic material, thereby relieving respiratory failure. The related clinical study also demonstrated that Chaiqinchengqi decoction could reduce gastrointestinal failure and the duration of respiratory failure in patients with acute pancreatitis (Deng et al., 2025). Although the DCHD group showed a more significant difference in reducing PT, APTT and LAC compared with the placebo group, there was no significant difference in changes from baseline between the two groups. Critically, concerns have been raised about the safety profile of certain herbal medicines, but no serious AEs associated with DCHD were recorded in our study. However, potential safety considerations may persist, as the inclusion of *Rhei Radix et Rhizoma* (Dahuang) in DCHD may induce diarrhea, potentially elevating the risk of hypokalemia. These AEs were readily resolved upon discontinuation of DCHD and implementation of appropriate symptomatic management. Notably, related research proposed that increased fecal output may signify enhanced intestinal motility and functional restoration (Deng et al., 2025).

Metabolomics is now extensively applied in research on the complex molecular mechanisms of TCM in disease treatment. It facilitates the elucidation of herbal formula mechanisms, evaluation of therapeutic efficacy, investigation of compatibility principles, and quality control. The flavonoid-derived DMs Wogonin, Wogonoside, and Tectoridin are likely bioactive circulating components contributing to the efficacy of DCHD, which aligns with our prior mass spectrometry findings (Huang et al., 2025b). Wogonin is a key metabolite derived from the *Scutellariae Radix* (Huangqin), exerts therapeutic effects in sepsis through multifaceted mechanisms encompassing anti-inflammatory, antioxidant activities, and modulation of signaling pathways. A study has demonstrated that in LPS-induced inflammatory models, Wogonin modulates SIRT1-mediated deacetylation of HMGB1 and suppresses the expression of pro-inflammatory cytokines including TNF-α, IL-1β, and IL-6 (Ge et al., 2021). Besides, Tectoridin has been shown *in vitro* and *in vivo* to mitigate LPS-induced inflammatory responses by inhibiting the TLR4/NF-κB/NLRP3 signaling pathway (Niu et al., 2022). Cholic acid represents one of the primary bile acids directly biosynthesized within the human liver. Conversely, glycocholic acid constitutes a conjugated bile acid derivative formed via the hepatic conjugation of cholic acid with the amino acid glycine. During sepsis, cholestasis and the accumulation of circulating bile acids constitute frequent early manifestations (Jenniskens et al., 2018). Research has demonstrated that Yinchen Decoction modulates hepatic and ileal FXR signaling, thereby ameliorating cholestasis-induced liver injury (Song et al., 2023). Systematic network analysis revealed that Dahuang Xiaoshi Decoction exerts its anti-cholestatic effect in alpha-naphthylisothiocyanate-induced cholestatic rats primarily by regulating primary bile acid biosynthesis, arginine and proline metabolism (Zhu and Feng, 2019). Notably, ORA analysis suggests that bile acid metabolism is one of the significantly enriched metabolic pathways. Previous animal studies have demonstrated that DCHD targets the PPARα signaling pathway governing bile acid metabolism, effectively alleviating liver injury induced by both α-naphthylisothiocyanate-triggered intrahepatic cholestasis and bile duct ligation-induced extrahepatic cholestasis (Wang et al., 2025; Zhou et al., 2022). Collectively, these findings suggest that active constituents of DCHD may ameliorate SLI by modulating bile acid metabolism, which is closely associated with DCHD’s established soothing liver-gallbladder (*shu gan li dan*) efficacy in TCM.

Our study also presents several limitations. Firstly, the single-center and single-blind design are limitations that affect the generalizability of our findings, and clinical practices for managing septic patients may vary across geographical regions and healthcare settings. Secondly, a comprehensive assessment of DCHD’s impact on cholestasis in SLI patients should include the evaluation of specific biomarkers, γ-glutamyltransferase (γ-GT) and alkaline phosphatase (ALP), supplemented by targeted metabolomics to elucidate the mechanistic underpinnings of bile acid metabolism. Furthermore, the relatively limited sample size underscores the urgent need for large-scale, multi-center clinical studies. Secondly, due to the critical and rapidly progressive nature of sepsis, blinding of the investigators was not implemented in this study. Moreover, the development of a credible placebo for complex herbal decoctions remains a persistent challenge in clinical trials of traditional Chinese medicine. Finally, although DCHD, as a classical herbal formula, has demonstrated clinical efficacy in this research, its active compounds and underlying molecular mechanisms warrant further elucidation.

## 5 Conclusion

Our findings provide promising evidence supporting the clinical efficacy of DCHD in the treatment of SLI. The preliminary results demonstrate that adjunctive DCHD therapy, administered in conjunction with guideline-directed therapy, significantly ameliorates hepatic function, infection status, respiratory parameters, and disease severity in SLI patients, although it did not reduce 28-day all-cause mortality. This therapeutic benefit is likely attributable to the modulation of bile acid metabolism. However, further validation of the safety and efficacy profile of DCHD for SLI management is warranted to implement through multicenter, double-blind, randomized controlled trials.

## Data Availability

The original contributions presented in the study are included in the article/Supplementary Material, further inquiries can be directed to the corresponding author.

## Glossary

SLI: septic liver injury
DCHD: Dachaihu Decoction
ALT: alanine transaminase
AST: aspartate transaminase
TBil: total bilirubin
SOFA: Sequential Organ Failure Assessment
APACHE II: Acute Physiology and Chronic Health Evaluation II
ICU: intensive care unit
TCM: Traditional Chinese Medicine
LPS: lipopolysaccharide
INR: International normalized ratio
AASLD: American Association for the Study of Liver Diseases
MCID: Minimal Clinically Important Difference
CRP: C-reactive protein;
PCT: procalcitonin
WBC: white blood cell
NEU: neutrophils
PT: Prothrombin Time
APTT: Activated partial thromboplastin time
LAC: lactic acid
OI: oxygenation index
AEs: adverse events
SD: standard deviation
IQR: interquartile range
BMI: body mass index
CRRT: continuous renal replacement therapy
ECMO: extracorporeal membrane oxygenation
IABP: intra-aortic balloon pump
ARDS: acute respiratory distress syndrome
AGI: acute gastrointestinal injury
DMs: differential metabolites
LC-MS/MS: Liquid Chromatography-Tandem Mass Spectrometry
PCA: Principal Component Analysis
PLS-DA: Partial Least Squares Discriminant Analysis
ORA: Over-Representation Analysis
FDR: False Discovery Rate
γ-GT: γ-glutamyltransferase
ALP: alkaline phosphatase
