# Supplementary material for: Clinical efficacy and metabolomics profiling of dachaihu decoction for patients with septic liver injury: a randomized controlled trial
Source: Front Pharmacol. 2025 Nov 25;16:1671732. doi: 10.3389/fphar.2025.1671732 (PMC12685928; doi:10.3389/fphar.2025.1671732)
Supplement: Supplementary file 2 [file Supplementaryfile1.pdf]

伦理委员会批件

批件号：KY【2024】024

<table>
<tr><td>项目名称</td><td colspan="4">基于“肠-肝轴”理论探究大柴胡汤对脓毒症肝损伤的影响及作用机制</td></tr>
<tr><td>项目来源</td><td colspan="4">研究生毕业论文</td></tr>
<tr><td>科室/专业</td><td colspan="2">急诊科</td><td>主要研究者</td><td>何明丰、杨震</td></tr>
<tr><td rowspan="3">审查情况</td><td>初始审查方式</td><td>会议审查</td><td>审查日期</td><td>2024-01-12</td></tr>
<tr><td>审查地点</td><td colspan="3">线上会议</td></tr>
<tr><td>表决情况</td><td colspan="3">参会 13 人，投票 13 人，回避 0 人；（详见会议出席表）
同意 13 票，修改后同意 0 票，不同意 0 票。</td></tr>
<tr><td>审查决定</td><td colspan="4">同意开展研究。</td></tr>
<tr><td>审查同意文件</td><td colspan="4">1. 初始审查申请
2. 研究方案(版本号:V3.0;版本日期:2023-12-19)
3. 知情同意书(版本号:V3.0;版本日期:2023-12-19)
4. 研究者专业履历
5. 研究生开题报告
6. 材料诚信承诺书</td></tr>
<tr><td>跟踪审查频率及批件有效期</td><td colspan="4">跟踪审查频率 12 个月，伦理委员会有根据实际进展情况改变跟踪审查频率的权利。
请于 2024 年 12 月中旬提交年度/定期跟踪审查报告（研究进展报告）。
批件有效期为：2024 年 1 月 12 日至 2025 年 1 月 11 日。</td></tr>
<tr><td>主任委员/被授权者签字</td><td colspan="4">[签名] 日期：2024 年 1 月 12 日</td></tr>
<tr><td>盖章</td><td colspan="4">佛山市中医院医学伦理委员会</td></tr>
<tr><td>声明：</td><td colspan="4">1. 佛山市中医院医学伦理委员会(以下简称本伦理委员会)的相关工作遵循中华人民共和国《涉及人的生物医学研究伦理审查办法》和《药物临床试验质量管理规范》等国家法律法规以及 ICH-GCP 的要求操作。
2. 研究实施前提：所有研究需经伦理委员会审查获得同意后方可实施，实施过程应遵循伦理委员会同意的方案执行，应符合赫尔辛基宣言和 GCP 的基本原则。医学研究应当在国家全民健康保障信息平台医学研究登记备案信息系统中进行备案。特殊情况：(1) 属《人类遗传资源采集、收集、买卖、出口、出境</td></tr>
</table>

	审批行政许可事项》规定范畴的研究，获得中国人类遗传资源管理工作办公室批准/备案后，将批准/备案成功结果书面递交伦理委员会备案后方可实施。(2)属《需进行临床试验审批的第三类医疗器械目录》内医疗器械的临床试验，获得国家药品监督管理局备案成功结果书面提交伦理委员会备案后方可实施。<br>(3)属需在国家药品监督管理局备案/默示许可的项目，获得备案成功结果/默示许可的结果及时提交伦理委员会备案后方可实施。<br>3. 研究过程中，对研究方案和知情同意书等相关文件所作的任何修订，均需得到本伦理委员会审查同意后方可实施。<br>4. 研究过程中，发生的以下情况需要及时报告伦理委员会：(1)在本中心发生的严重不良事件（SAE）或可疑且非预期严重不良反应（SUSAR）以及药物安全报告评估摘要等安全性信息；(2)本中心发生的严重或持续的方案违背/偏离；(3)暂停或终止研究。<br>5. 需按照伦理初始审查伦理批准函的持续审查频率和首次批准时间提交持续审查申请，确保批件到期前 1 个月递交年度跟踪审查申请，以获得伦理委员会的批准。(本伦理委员会有权根据实际开展情况改变持续审查频率)<br>6. 本中心研究结束时，需在关闭中心前及时向本伦理委员会递交研究完成报告及相关附件。

联系方式：广东省佛山市禅城区亲仁路 6 号佛山市中医院　邮编：528000
电话：0757-83068422/83067111　邮箱：fstcmmec@fshtcm.com.cn
