# Supplementary material for: Clinical efficacy and metabolomics profiling of dachaihu decoction for patients with septic liver injury: a randomized controlled trial
Source: Front Pharmacol. 2025 Nov 25;16:1671732. doi: 10.3389/fphar.2025.1671732 (PMC12685928; doi:10.3389/fphar.2025.1671732)
Supplement: Supplementary file 3 [file Supplementaryfile3.docx]

**Supplement 2**

**临床试验随机分组表**

**Clinical trial randomization schedule**

| Visit number | Random number | Group | Visit number | Random number | Group | Visit number | Random number | Group |
| --- | --- | --- | --- | --- | --- | --- | --- | --- |
| 就诊序号 | 随机数 | 分组 | 就诊序号 | 随机数 | 分组 | 就诊序号 | 随机数 | 分组 |
| 1 | 1.40 | 1 | 29 | 0.53 | 1 | 57 | 3.47 | 1 |
| 2 | 4.31 | 2 | 30 | 7.38 | 2 | 58 | 9.21 | 2 |
| 3 | 6.12 | 2 | 31 | 1.20 | 1 | 59 | 4.08 | 2 |
| 4 | 2.91 | 1 | 32 | 0.59 | 1 | 60 | 0.96 | 1 |
| 5 | 1.56 | 1 | 33 | 1.88 | 1 | 61 | 3.34 | 1 |
| 6 | 7.00 | 2 | 34 | 1.00 | 1 | 62 | 6.21 | 2 |
| 7 | 3.46 | 1 | 35 | 6.76 | 2 | 63 | 6.41 | 2 |
| 8 | 4.46 | 2 | 36 | 6.93 | 2 | 64 | 7.52 | 2 |
| 9 | 0.52 | 1 | 37 | 8.67 | 2 | 65 | 2.05 | 1 |
| 10 | 1.03 | 1 | 38 | 3.51 | 1 | 66 | 1.43 | 1 |
| 11 | 1.41 | 1 | 39 | 4.96 | 2 | 67 | 4.46 | 2 |
| 12 | 0.43 | 1 | 40 | 7.02 | 2 | 68 | 3.69 | 1 |
| 13 | 6.22 | 2 | 41 | 2.06 | 1 | 69 | 4.85 | 2 |
| 14 | 1.54 | 1 | 42 | 7.73 | 2 | 70 | 0.99 | 1 |
| 15 | 7.15 | 2 | 43 | 0.67 | 1 | 71 | 7.26 | 2 |
| 16 | 9.28 | 2 | 44 | 5.27 | 2 | 72 | 0.37 | 1 |
| 17 | 5.78 | 2 | 45 | 6.62 | 2 | 73 | 1.75 | 1 |
| 18 | 2.62 | 1 | 46 | 9.36 | 2 | 74 | 2.88 | 1 |
| 19 | 7.25 | 2 | 47 | 3.16 | 1 | 75 | 6.69 | 2 |
| 20 | 0.37 | 1 | 48 | 5.23 | 2 | 76 | 7.61 | 2 |
| 21 | 1.01 | 1 | 49 | 7.66 | 2 | 77 | 8.03 | 2 |
| 22 | 7.33 | 2 | 50 | 1.27 | 1 | 78 | 7.03 | 2 |
| 23 | 2.25 | 1 | 51 | 5.59 | 2 | 79 | 0.48 | 1 |
| 24 | 2.21 | 1 | 52 | 9.83 | 2 | 80 | 3.94 | 1 |
| 25 | 6.10 | 2 | 53 | 3.25 | 1 | 81 | 2.29 | 1 |
| 26 | 9.54 | 2 | 54 | 5.36 | 2 | 82 | 7.26 | 2 |
| 27 | 2.37 | 1 | 55 | 5.08 | 2 | 83 | 8.82 | 2 |
| 28 | 3.39 | 1 | 56 | 2.82 | 1 | 84 | 1.82 | 1 |

注：起点设置为2000000；随机数设置为1-10；分组中1代表安慰剂组；2代表治疗组

Note: The starting point is set to 2000000; the random number is set to 1-10; 1 represents the placebo group and 2 represents the treatment group
