# Supplementary material for: Clinical efficacy and metabolomics profiling of dachaihu decoction for patients with septic liver injury: a randomized controlled trial
Source: Front Pharmacol. 2025 Nov 25;16:1671732. doi: 10.3389/fphar.2025.1671732 (PMC12685928; doi:10.3389/fphar.2025.1671732)
Supplement: Supplementary file 4 [file Supplementaryfile4.docx]

**Supplement 4 Fingerprint analyses of DCHD
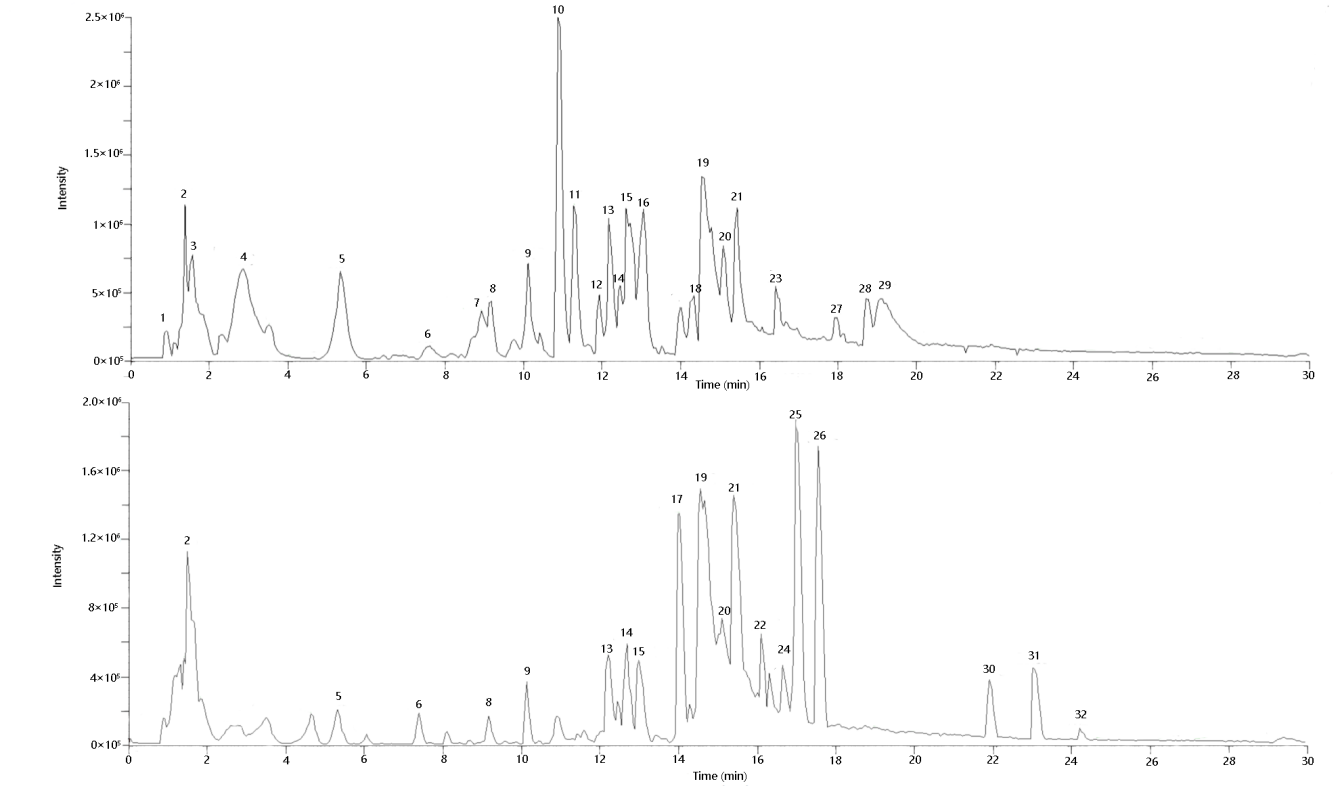
**

1.2-Hydroxyhippuric acid, 2.Trigonelline, 3.Theophylline, 4.Citric acid, 5.L-Phenylalanine 6.3-Ethyl-4-hydroxy-1-phenyl-1,2-dihydroquinolin-2-one, 7.Coniferin, 8.Salicylic acid, 9.Albiflorin, 10.Quercetin, 11.Esculetin, 12.Hesperetin, 13.Genistein, 14.Naringin, 15.Kaempferol, 16.Nictoflorin, 17.Neohesperidin, 18.6-O-Methylscutellarin, 19.Baicalin, 20.Apigetrin, 21.Wogonin, 22.Obacunone, 23.Tangeritin, 24.Rubiadin, 25.Shogaol, 26.Nobiletin, 27.Saikosaponin A, 28.Genistein, 29.Daidzein, 30.Muscone, 31.1-Stearoylglycerol, 32.Emodin
